# A qualitative approach to assess the opinion of physicians about the challenges and prospects of pharmacogenomic testing implementation in clinical practice in Greece

**DOI:** 10.1186/s40246-024-00648-y

**Published:** 2024-07-19

**Authors:** Margarita-Ioanna Koufaki, George P. Patrinos, Konstantinos Z. Vasileiou

**Affiliations:** 1 Department of Pharmacy, Laboratory of Pharmacogenomics and Individualized Therapy, University of Patras School of Health Sciences, University Campus, Patras, GR-26504 Greece; 2 College of Medicine and Health Sciences, Department of Genetics and Genomics, United Arab Emirates University, Al-Ain, Abu Dhabi, United Arab Emirates; 3 Zayed Center for Health Sciences, United Arab Emirates University, Al-Ain, Abu Dhabi, United Arab Emirates

**Keywords:** Pharmacogenomics, Physicians, Semi-structured interview, Attitudes, Perceptions, Intentions to adopt, Qualitative research, Future recommendations

## Abstract

**Background:**

Pharmacogenomics (PGx) constitutes an important part of personalized medicine and has several clinical applications. PGx role in clinical practice is known, however, it has not been widely adopted yet. In this study, we aim to investigate the perspectives of Greek physicians regarding the implementation of PGx testing in clinical practice and the key issues associated with it.

**Methods:**

Fourteen interviews were conducted with physicians of various specialties for which PGx applications are available. A semi-structured interview guide was utilized based on the Consolidated Framework for Implementation Research (CFIR) context and the Diffusion of Innovation model. Transcripts were coded independently and compared by two members of the research team. Descriptive statistics were generated using Microsoft Excel.

**Results:**

Six main themes emerged: awareness and use of PGx testing; source of information; key stakeholders of the PGx supply chain, their interactions and change agents; clinical benefit and significance of PGx testing; barriers and lack of reimbursement; and recommendations to boost the PGx adoption rate. Most respondents were aware of PGx applications, but only three had already recommended PGx testing. Peer-reviewed journals along with clinical guidelines were regarded as the most used source of information while stakeholders of the PGx supply chain were discussed. PGx was considered that promote patient-centered care, enhance medication clinical effectiveness, decrease the risk of side effects, and reduce healthcare costs. Lack of reimbursement, scarcity of resources, and high PGx cost were the foremost barriers affecting PGx adoption.

**Conclusions:**

It was concluded that if case PGx testing is reimbursed and physicians’ training is reinforced, PGx implementation will be boosted and improved shortly.

**Supplementary Information:**

The online version contains supplementary material available at 10.1186/s40246-024-00648-y.

## Introduction

Currently, personalized medicine interventions, particularly pharmacogenomics (PGx) are a significant trend in the healthcare field [1, 2].

Over the last two decades, receiving tailored-made services ranging from advertisement to medication is an emerging tendency that has affected all aspects of our life. “One size fits all” has been slowly abandoned as a model given that our knowledge of genetics is getting deeper and deeper, a fact that allows us to move towards personalized medication treatment [3].

Researchers highlight that each individual has unique characteristics in terms of his/her response to medications, and that is crucial to provide a more tailored therapeutic strategy to deal with interindividual variance, avoid adverse drug reactions (ADRs), and get greater clinical effectiveness in a shorter time. To do so, PGx testing is recommended by several research networks (Clinical Pharmacogenetics Implementation Consortium (CPIC), Dutch Pharmacogenetics Working Group (DPWG)) and regulatory bodies including the European Medical Association (EMA), and the Food and Drug Association (FDA) [1]. According to Shin and coworkers, 2009, the FDA considers PGx as a way to find new biomarkers involved in drug development and induce the field [4], while since 2012, the EMA has launched an initiative to make PGx drug label visible to healthcare professionals and inform them [5].

Nonetheless, even if there is sufficient clinical evidence from recent studies [6] and established guidelines to support the advantages of preemptive PGx testing in determining a patient’s therapy, the PGx testing adoption rate among physicians is still low, and it shows great variability among countries [7]. Regulatory agencies, research associations, and working groups have established programs and seminars aiming to induce the adoption of PGx in the clinical setting by either focusing on physicians’ education and training or by providing new and innovative tools such as decision-making applications and supporting guidelines to help them and increase their self-confidence [1, 5].

According to Chenoweth and coworkers, 2020, ten main challenges impede PGx testing’s widespread implementation in clinical practice, with two focusing on the role of the physicians’ community and its attitude towards PGx [8]. These observations are supported by many quantitative and qualitative studies that evaluate healthcare professional perceptions and attitudes. Based on Koufaki and coworkers, 2021, prospective PGx adoption confronts several challenges and obstacles in being introduced in clinical practice [9]. Different systematic reviews on the topic concluded that physicians have a low level of awareness, moderate self-confidence, and a lack of practical knowledge related to genomic testing [10], and in particular in PGx testing [11, 12].

To date, there are only a few qualitative studies in the field, and most of them are performed in Western countries and mostly in the US. There is a significant lack of information about the opinions and perspectives of physicians in European countries about PGx where the structure of the healthcare system is different, medication reimbursement is limited, and the physician in most cases is the only healthcare professional with the right to prescribe a medication. Moreover, in the existing literature, a significant part of researchers had focused on one specialty of physicians each time and had not included all of the specialties for which there are available PGx testing applications [10, 11]. Given that physicians are key stakeholders with the right to prescribe medications [12], it is obvious that their contribution to PGx adoption is essential and decisive. Understanding physicians’ perceptions, opinions, and attitudes, particularly before implementing PGx in clinical practice can contribute to better outcomes and marketing strategies.

In this study, we aim to investigate and showcase the perspectives of Greek physicians who specialize in different therapeutic areas and work in different hospitals across the country regarding the implementation of PGx testing in clinical practice and the key issues associated with it. In Greece, PGx testing is available and it is offered by several public or private laboratories. In this study, we examined PGx as a whole discipline, in line with a great part of relevant prior quantitative and qualitative research [13–16]. By performing one-to-one interviews we had the objective of highlighting their thoughts and suggestions on what factors affect their willingness to adopt PGx testing in their daily practice.

## Material & methods

### Study cohort

The study focused on physicians with specialties for which PGx applications are available based on regulatory agencies’ recommendations. To obtain diverse perspectives in the field, a purposeful sampling plan following the snowball approach was applied; physicians were recruited from various hospitals, educational backgrounds, professional levels, etc., and were interviewed between 15 January 2024 and 1 March 2024. A total of 25 physicians were invited via email to participate in the study. Physicians invited were found via networking and referrals of other colleagues and experts in the field. Recruitment was an ongoing process until data saturation occurred, namely when sufficient data was collected to achieve the study objectives, as the last few interviews provided no new themes and rather similar explanations. Physicians’ specialties included oncologists, cardiologists, general practitioners, psychiatrists, nephrologists, gastroenterologists, and dermatologists. The choice of these specialties was based on the existing literature about current PGx clinical applications [2]. Invited physicians were working both in the public and private sectors across different areas of Greece, with most of them being in Athens, the capital of Greece.

Physicians who agreed to participate were provided with a list of questions a day before the interview. The semi-structured interview was conducted mostly via online meeting that lasted for approximately 40 min. Self-reported demographic data were also obtained at the beginning of each interview. The Institutional Ethical Board of the University of Patras approved the conduction of the study with approval number 16,383/29.02.2024 and participants provided their informed consent in writing by responding to the invitational email.

### Data collection & analysis

A semi-structured interview guide was developed and included a set of 21 open-ended questions classified into 6 different factors as described in the supplementary material. The included factors were based on the Consolidated Framework for Implementation Research (CFIR) context and the diffusion of innovation model [17–19].

Incorporated factors were also confirmed with current evidence from recent systematic reviews in the field highlighting the most important factors that affect physicians’ intentions to adopt genomics or PGx applications in clinical practice [20–22]. More precisely, the questions were grouped into 7 different sections and covered the following topics: (A) awareness and use of PGx testing; (B) sources of information about PGx; (C) key stakeholders of the PGx supply chain and their interactions; (D) change agents in the PGx supply chain; (E) benefits and usefulness of PGx testing; (F) barriers of PGx implementation and lack of reimbursement of PGx testing; (G) future prospects and recommendations to boost the PGx adoption rate.

Interviews were audio-recorded and transcribed verbatim by Google Live Transcribe. Deidentified transcripts were analyzed using a hybrid of inductive and deductive coding followed by theme development according to CFIR guidance. In the beginning, all results were documented in an Excel sheet and an initial code book with concepts was created as part of the deductive portion to categorize interviewees’ responses [23–25]. This elucidated approximately 70% of the final codes. New codes were added to the final codebook for additional concepts highlighted by participants. All coding process was performed at the paragraph level to capture full responses to interview questions. No coding application was used because the available options in the Greek language were limited and their transcript quality was weak. Transcripts were coded independently and compared by two members of the research team (MIK, KZV). Any differences were resolved through discussions aimed at reaching a consensus, followed by rehearing of applicable sections of the transcripts. Descriptive statistics were generated using Excel.

## Results

Of the 25 physicians who were contacted via email, 14 (56%) agreed to participate in an interview. A description of the study cohort is demonstrated in Table 1. The cohort consisted of general practitioners (*n* = 4), oncologists (*n* = 3), psychiatrists (*n* = 3), nephrologist (*n* = 1), cardiologist (*n* = 1), gastroenterologist (*n* = 1), and dermatologist (*n* = 1). The group was balanced in terms of gender, while the mean cohort age was 45 years old. Two-thirds of the participants were working in a public hospital. All participants had a medical degree (MD). Additionally, one of them held an MSc title and six had a PhD title. Participants had been in practice for 1–5 years (*n* = 4), 6–10 years (*n* = 2), 11–20 years (*n* = 7), and more than 20 years (*n* = 1).

**Table 1:** Participants demographics

Age	
**25–30 years old**	2
**31–45 years old**	5
**46–60 years old**	5
**61–65 years old**	2
**Gender**	
Female	7
Male	7
**Specialty**	
Oncologists	4
Psychiatrists	3
General Practinioners	3
Dermatologist	1
Cardiologist	1
Nephrologist	1
Gastroenterologist	1
**Years as a practicing physician**	
1–5 years	4
6–10 years	2
11–20 years	7
> 20 years	1
**Academic Rank**	
MD only	7
MSc	1
PhD	6
**Formal education or training in PGx**	
Yes	3
No	11
**Past Experience with PGx testing**	
Yes	5
No	9

Moreover, qualitative analysis of the interviews revealed six major themes influencing physicians’ perceptions about PGx testing in clinical practice; Awareness and use of PGx testing, Sources of information, Key stakeholders of the PGx supply chain, their interactions, and the Change agents, Benefits & Usefulness of PGx testing, Barriers of PGx implementation and Lack of reimbursement of PGx testing, Future prospects and Recommendations to boost the PGx adoption rate. Key points for each topic are shown in Table 2.

**Table 2:** Summary of the study’s findings in key points

Theme	Subtheme
Awareness & Use of PGx testing	- Most participants haven’t attended any session or course on the PGx topic. - Five out of 14 have recommended PGx testing once. - All participants considered that PGx has clinical application in their specialty. - No respondent knew any colleague who had already recommended PGx testing so far. - There is a lack of available clinical guidelines from Greek bodies. Most of them are derived from international institutions.
Sources of information	- Peer-reviewed publications, clinical guidelines from official bodies, conferences & congresses are the main sources of information. - The business-to-customer marketing approach is not available in the PGx market. - There are no well-developed communication channels.
Key stakeholders of the PGx supply chain, their interactions and Change agents in the PGx supply chain	- There are many stakeholders involved in the process: patients, physicians, laboratory site personnel, patients’ caregivers, pharmacists/ clinical pharmacists, pharmaceutical companies, local healthcare authorities, insurance bodies or companies and the hospitals’ administration - Key interactions among stakeholders: physicians with patients, laboratory units, hospital administrators, patient caregiver, clinician pharmacists, pharmaceutical companies, regulatory bodies and other public authorities; local and global regulatory bodies with other stakeholders although not directly communicating with them, - Change agents and their interactions: physician due to direct contact with patients and desire to better fulfill their needs, and interactions with all key stakeholders in the PGx supply chain; laboratories to increase their revenues; public authorities, health insurance (public & private) and hospital managers to reduce spending and improve clinical outcomes; pharmaceutical companies to increase revenues and market share - Regulatory/public authorities, physicians, and pharmaceutical companies are the stakeholders with greater strength and influence in the PGx supply chain.
Benefits & Usefulness of PGx testing	**Personalised treatment** - Patient-centered approach. - Tailormade treatment scheme. **Clinical effectiveness**: - Fewer hospital admissions. - Reduced risk of ADR occurrence. - Greater clinical effectiveness. - Avoidance of dose titration. **Social Impact**: - Patients experience a better quality of life due to fewer ADRs. - The risk of medical errors occurrence is decreased. - Patients have better mental health / higher level of treatment satisfaction. - Increase of patient adherence to recommended treatment - Amelioration of physician-patient relationship - Physician feels more confident/secure for the recommended treatment **Economic impact**: - Reduction of healthcare expenditures especially for medications. - Fewer hospitalization days.
Barriers of PGx implementation and Lack of reimbursement of PGx testing	**Resources Related**: - Scarcity of specialized human resources. - Lack of available laboratories and infrastructures. **Bioethics**: - Bioethical concerns. - Data protection and confidentiality. **Testing Features**: - High cost of PGx testing. - Lack of PGx testing reimbursement. - Time-consuming procedure. **Physicians**: - Lack of clinical guidelines. - Low level of awareness and knowledge among physicians. - Lack of motivation to recommend PGx testing. **Patients**: - Social prejudice about PGx testing. - Psychological distress. - Lack of information among patients. - Patients’ disbelief towards new technologies. **Lack of reimbursement**: - High PGx testing cost. - Lack of financial resources. - Limited clinical data about PGx cost-benefit. - No official healthcare policy for PGx adoption. - PGx testing is not mandatory.
Future prospects and Recommendations to boost the PGx adoption rate	**Future prospects**: A slow but steady increase is anticipated for the PGx adoption rate. **Recommendations**: **Clinical Evidence**: - Promote clinical research in the field to get more clinical evidence. Researchers, laboratories, and other research institutions can be change agents in PGx adoption. - Improve clinical guidelines for physicians. **Cost Reduction**: - Reimburse PGx testing. - Reduce PGx testing cost. **Physicians**: - Raise awareness and train physicians in PGx. - Get involved in the promotion of PGx testing. **Social policy**: - Change the legal framework for genetic testing. - Create better communication channels among stakeholders and enhance their interactions. **Patients**: - Raise awareness about PGx testing in patients.

### Awareness and use of PGx testing

Most of the respondents had heard about PGx and its clinical application except for two who did not characterize themselves as aware of this technology. In that case, an explanation of PGx term was made and it was ascertained that physicians got confused with genomics and PGx. However, all agreed that PGx testing is applicable in their specialty and that there are clinical applications available to use. An oncologist mentioned that “*There are clinical projects in which cancer patients can participate and get their PGx profile*”, while a psychiatrist stated that “*A few years ago*,* I had participated in a European*,* multisite study focused on the preemptive PGx testing and more than 1300 patients with mental disorders were enrolled in the study.”* The vast majority of respondents had not attended any PGx-related session or lecture during their undergraduate or postgraduate studies except for three respondents. Moreover, when asked whether they had recommended a PGx test to a patient in the past, five out of 14 answered positively, stating that they had prescribed a PGx test more than once. On the contrary, respondents claimed that they did not know any colleague who had recommended such testing. A physician also commented, *“My fellow psychiatrists did not recommend them because either they are not convinced about their clinical effectiveness based on the available data or they just underestimate their clinical significance.”*

Furthermore, it was underlined that there is an important lack of clinical guidelines about PGx in most specialties. Only 6 out of 14 were aware of available guidance in their field. More precisely, all participated oncologists shared examples of global organizations (i.e. FDA), medical associations, or societies that had already published relevant material for the application of PGx testing, while two psychiatrists provided more specific examples based on their experience with PGx testing.

A psychiatrist claimed: *“Clinical guidelines on the topic are available. They mostly explain how to adjust a patient’s treatment based on his/her results. There is also a book on the application of PGx testing in psychiatry. I was the editor of the book so I am aware of it. More publications*,* guidelines*,* and bibliography are coming on the topic to satisfy physicians’ needs.”*

Nevertheless, it was concluded that most of the available clinical protocols and guidelines are not created at a national level but they derived from international bodies, implying that there is no available material written in the Greek language or being approved by local authorities. Besides the aforementioned lack, a physician mentioned that *“physicians are not dictated to comply with the guidelines or commit to them since the guidelines are not mandatory and leave the final decision at the physician’s discretion.”*

### Sources of information

Based on physicians’ feedback, the most valuable and useful source of information is scientific publications in international peer-reviewed journals followed by regulatory bodies (FDA, EMA), international medical associations, patient societies, and participation in conferences and congresses. In particular, respondents were shown to trust official authorities’ guidelines, announcements, and memos for their proper information. It is common to get newsletters or emails from medical associations for updates.

It was also highlighted that there is no communication or any form of interaction with salespersons or sales forces of pharmaceutical companies or laboratories that offer PGx testing services. Self-advancement is mainly the reason why physicians want to get updated about new trends on the topic since the applicable sources of information and communication channels seem not to be well-developed yet for PGx testing compared to other genomic products. One oncologist stated that: *“We don’t get informed by any lab. As a physician*,* I can give a call to a lab and ask them about their PGx services. However*,* this is not common. Only if a physician believes in the benefit of PGx and he/she is convinced about the usefulness of PGx results to patient healthcare management*,* they will reach out to the lab.”*

***Key stakeholders of the PGx supply chain***,*** their interactions***,*** and Change agents.***

When participants were asked to name and classify the most prevalent stakeholders involved in the PGx process, they all included the most basic stakeholders involved in the PGx supply chain; patients, physicians, laboratory site personnel including biologists, geneticists, technicians, and genetic counselors. We define basic stakeholders based on Mitropoulou and coworkers, 2014 and Rahma and coworkers, 2021 [26, 27]. Some of them expanded stakeholders’ supply chains. They incorporated other groups such as patients’ caregivers, pharmacists/ clinical pharmacists, and producers (i.e. pharmaceutical companies, sales representatives) along with local authorities (i.e. ethical committees, health technology assessment agencies, reimbursement committees, ministry of Health, regulatory bodies), insurance bodies or companies and the hospitals’ administration. Admittedly, most physicians lacked the wider picture of stakeholders and only those with previous experience with PGx testing could identify more stakeholders.

The interactions among stakeholders were also investigated. It was found that physicians are directly connected and interact with patients since they are their main point of contact. Physicians recommend PGx testing and determine the drug and disease management for each individual. In parallel, physicians are closely collaborating with laboratory units to proceed with the test and gain expertise by lab-based specialties. Finally, hospital administrators are the physicians’ employers so they exert an impact on them and allocate the available resources. Hospital administration is an important stakeholder based on physicians’ opinions because it makes the necessary decisions for the induction of new technologies or testing within hospital infrastructures.

Moreover, it was thoroughly pinpointed the need for a legal parameter for the proper procedure of the PGx flowchart. Local and global regulatory bodies are responsible for the accreditation and validity of PGx testing, providing clinical guidelines and requirements, setting the framework for PGx completion, and approving the launch of a series of PGx products in the market. It seems that these institutions are not directly communicating with the other stakeholders, but they affect them with their decisions. Other governmental bodies i.e. Ministry of Health and, ethical committees share some common features with regulatory authorities’ role in the supply chain and also impact on the rest of the stakeholders following our results.

Finally, it was mentioned that there are reimbursement committees, public or private insurance organizations, and of course pharmaceutical companies involved in the PGx supply chain. Reimbursement committees and insurance organizations are related to the financial aspect of PGx testing. Their verdict can decisively change the route of PGx testing and they are in close communication with physicians who are the experts in the field. It was also commented that pharmaceutical companies may be actively involved in the advertisement and promotion of testing among the healthcare community via salespersons and be a valuable source of information for physicians.

Apart from the PGx supply chain, respondents were asked to highlight the change agents present in the PGx field in Greece, namely the stakeholders with a strong incentive to boost PGx adoption in daily clinical practice. Based on their answers, it was found that there are different perceptions about change agents among physicians. More precisely, some of them concluded that physicians are the change agents for PGx testing because they are the opinion experts and they represent their patients to the rest of the stakeholders. Their attitude can affect all decision-makers since *“Physician has a pivotal position in it since he communicates and interacts with all stakeholders involved. The most important part is that the physician has easy access to the end user that is the patient.”*, a psychiatrist added.

Our data show that many change agents have the power and the incentive to enhance the presence of PGx applications. Laboratories have a strong motive to be a change agent and establish PGx testing in the market because they perform the testing and the results. Pharmaceutical companies are thought by some physicians that be strong change factors as well. It is believed that they have the strongest financial motive to boost PGx adoption in the clinical setting while they collaborate with most of the stakeholders as described above. Pharmaceutical companies were demonstrated to have another advantage. They know the market and have a well-structured team of experienced sales force that can support physicians’ and patients’ information on the topic. Indeed, *“Physicians’ perception and attitude towards a new technology can determine technology’s success and penetration in the market. Educating and training physicians in PGx is the key to success*,* and to do so*,* you need a well-trained sales force with good communication channels.”.*

Physicians are shown to be valuable stakeholders with strong interactions with all other stakeholders especially with patients. Being the representatives of patients, they can manage to exert an impact on decision-makers because they interact with public authorities, health insurance companies, and hospital managers to improve clinical outcomes while health spendings are reduced. In general, respondents mentioned that regulatory/public authorities, physicians, and pharmaceutical companies are considered the stakeholders with greater strength and influence in the PGx supply chain.

### Benefits & usefulness of PGx testing

All participants were aware of the importance of PGx testing in clinical practice and highlighted several important aspects related to it. Their comments were grouped into four big sub-theme categories including personalized medicine, clinical effectiveness, social impact, and economic impact. 11 out of 14 of the respondents stated that the PGx application can enhance the role of personalized medicine and provide tailored therapeutic options. *“Medicine is getting patient-centered this way”*, a participant mentioned.

The aspects of PGx clinical effectiveness were thoroughly discussed by respondents. Oncologists, in particular, highlighted the need to find the most suitable treatment for their patients as fast as possible since every day counts, implying that PGx implementation can reduce the trial and error period in stabilizing a patient’s treatment along with medication titration. According to them, cancer patients are fragile and frightened, so the treating physician wants to act immediately and reduce their exposure to toxic drugs. Moreover, according to all respondents, PGx testing can reduce the risk of ADRs occurrence and their repercussions. A psychiatrist mentioned *“I am trying to create long-lasting and stable relationships with my patients that rely on honest communication. When I prescribe a medication to them*,* I want to feel sure that it is safe for them and that they will not experience any reduced clinical effects or unpleasant effects. In this way*,* I can maintain a good relationship with them.”*

Many interviewees also pinpointed that the number of hospitalization days along with the duration of hospitalization may decrease because stratified treatment can have greater effectiveness, and improve patients’ adherence to therapy. Finally, two physicians described another aspect of PGx’s contribution to clinical practice and the healthcare sector. It was mentioned that *“Knowing the right medication for the right patient from the beginning can ameliorate the situation of overprescribing medications and deal with the scarcity of available medications in the market”.* Based on a cardiologist, patients suffering from cardiovascular disorders are usually under many medications and deal with polypharmacy.

Participants also highlighted another perspective of PGx related to its impact on individuals. Many physicians underlined the effect of PGx on a patient’s quality of life and overall experience because it is improved. As one physician stated, *“PGx makes me feel safer when I prescribe a medication to a patient because it reduces the risk of making a wrong choice and committing a medical error”.* Furthermore, PGx can have a financial impact. Based on respondents, preemptive PGx testing can reduce healthcare expenditures for patients’ hospitalizations, medications, and other procedures due to unpleasant ADRs and reduce the working hours of specialists.

### Barriers to PGx implementation and lack of reimbursement of PGx testing

Based on respondents’ comments lack of reimbursement by public or private insurance bodies was the foremost barrier that impedes PGx adoption in the clinical setting followed by the high cost of tests, timely procedure, and the low level of physicians’ knowledge. *“If a test can be prescribed then patients consider it as the most simple and natural thing in the world to do it. When a test is new and is not reimbursed*,* then patients don’t believe that it is accurate*,* they feel insecure and think that it is not official”*, said one specialist.

However, most of them were certain that *“Patients* will *perform PGx testing and pay it out of pocket. They will do what is best for their lives and will follow physicians’ recommendations”*. Indeed, one physician underlined *“Patients will conduct the test besides its high price if they understand the importance of the PGx results for their treatment. If a physician fails to convince his/ her patients about the PGx benefits*,* in combination with its high cost and the lack of reimbursement*,* no patient will perform it. Patients feel secure if corresponding authorities approve a new technology and it is reimbursed. In that case*,* the new intervention gains prestige in the eyes of the general public.”* Another specialist also added *“I would insist on a PGx testing for a patient that would benefit from it even if I knew that there might be financial obstacles. My ultimate goal as a physician is to grant patients access to new and innovative therapies.”* Moreover, only a few physicians (*n* = 4) expressed that available infrastructures and lab personnel were not adequate to support such technologies. The same respondents added that the PGx testing process can be too burdensome and lengthy, a fact that is associated with delays in results reporting. *“Cancer patients have no time to lose. They need to get their results as soon as possible. Getting their results in the given time is crucial for disease management and allows physicians to find alternatives”*, declared two oncologists.

A few respondents did touch upon other barriers also including bioethical issues, psychological distress of patients, social discrimination, and prejudices. For example, one oncologist commented on bioethics, data privacy, and confidentiality and clarified that: “*Bioethics or data confidentiality are not obstacles because the patient is informed about the test and informed consent can be signed between physician and patient. In Greece*,* cancer patients have a separate and cancer-stratified registry in which their data are anonymized and only authorized oncologists have access. Therefore*,* we ensure that important information is shared in real-time and that patients can get the best treatment in any hospital across the country*”.

However, in contrast with the above, another physician claimed that he was worried about the use of genetic data and he said *“As a citizen*,* I worry about PGx data privacy and confidentiality. It is easy to have a data breach and this data to be given to insurance companies.”*

Patients’ lack of information about PGx testing applications along with the psychological distress that can be caused by testing results was also noted. An oncologist said: “*Many patients don’t want to know the results of genetic testing. They asked us not to provide them with the results because they cannot deal with the psychological burden of having inherited a “bad”/ pathological gene from their descendants.”* Moreover, it is worth mentioning that the low level of physicians’ knowledge in combination with the lack of clinical guidelines are also impactful burdens based on participants’ feedback. *“Most physicians are not familiar with PGx testing and the lack of clinical guidelines and evidence to support a clinician’s decision prevent them from recommending a PGx testing”*, stated a participant.

Furthermore, when specialists were asked about the reason why PGx testing is not reimbursed, 8 out of 14 respondents claimed that their high cost could be the reason while some of them gave another perspective by implying that there might be a lack of sufficient clinical evidence to support PGx effectiveness. All agreed that the Greek healthcare system and professionals are not ready to embrace and adopt such pioneering technologies since physicians need better training and information on the topic. As they stated, there is no particular healthcare policy or initiative that promotes PGx testing at a national level. According to them, *“it is important to incorporate genetic testing in the designated long-term healthcare policy and not promote it as a short-term initiative to get a better adoption rate.”*

### Future prospects and recommendations to boost the PGx adoption rate

PGx testing will be further adopted in Greece based on our participants’ feedback. Most of the respondents (*n* = 13) claimed that the PGx adoption rate will be tripled within the next ten years reaching 15% of the market. Nonetheless, a slow but steady increase is anticipated, and the implemented changes won’t be remarkable. To do so, more clinical evidence about PGx effectiveness is required along with better clinical guidelines and support systems according to the majority of participants. As it was pinpointed, “*the launch of new clinical studies and research projects will better unfold the function of pharmacogenes*,* and drug-drug interactions and will put an added value in pharmacology. Having more clinical data and significant evidence about the effectiveness of PGx in the drug management of cancer or other rare diseases will improve the PGx adoption rate and will increase physicians’ readiness to implement such preemptive testing in their daily routine*”.

Moreover, some respondents believed that informing and training physicians about PGx applications would enhance PGx adoption. Specifically, it was claimed by a psychiatrist, *“The physicians who don’t recognize the advantages of PGx are those who are either afraid of the innovative and pioneer features of PGx*,* or those that had underestimated PGx’s usefulness. In both cases*,* the physician has a low level of knowledge about PGx and personalized medicine and he/she is not willing to keep up with global medical trends”.* Other respondents also noted that raising awareness of PGx testing among patients is highly important for wider adoption. *“Patients are sometimes more informed than physicians. Patients showed up in my office with publications and were asking for genetic testing. For me*,* it is crucial to have patients already familiar with the idea of PGx testing.”*, said an oncologist.

The reduction of PGx testing cost along with its reimbursement by public authorities was mentioned by all participants and it was found to be a decisive parameter for PGx adoption. *“By the time PGx testing is reimbursed*,* physicians have no reason not to recommend it to a patient.”* In addition to that, many respondents considered that the test should be offered by all public hospitals to enhance its role, accessibility, and validity. For this reason, it is crucial to increase resources (i.e., human resources, funding, lab equipment, etc.) in public hospitals and research labs. Respondents say, *“Hospitals are in great need of funding due to scarcity of resources*,* and physicians working in the hospital have hard-working hours and are overloaded.”* However, these changes also need updates in legislation and amendments in the Greek legal framework about such technologies. This was stated by a few respondents.

## Discussion

PGx is an emerging and promising technology that can change drug management of many chronic diseases including cardiovascular disorders, cancer, mental disorders, etc., and improve healthcare outcomes via better medication efficacy, fewer ADRs, and higher patient adherence. PGx testing is slowly adopted by countries across the globe and this is also the case in Greece. Based on the literature, many reasons affect PGx adoption in each country including physicians’ attitudes, social norms, perceived barriers, and self-confidence to implement PGx testing.

Based on our results, the vast majority of physicians had not attended any PGx-related course and only five of them had recommended or used PGx testing in the past. These findings are congruent with the literature. According to Ahmed and coworkers (2020), 56% of Jordanian physicians had heard of PGx terms and 32% believed that they have good knowledge [16]. In the Carroll and coworkers (2016) study, interviewed physicians had limited knowledge about the topic and the availability of PGx services [14]. Furthermore, in the qualitative study by Deininger and coworkers, 2019, 88% of cardiologists had not received any formal training but had been informed about PGx applications in some way [10]. In contrast, in China, it seems that PGx testing is widely applied since almost 60% of Chinese physicians have already recommended PGx testing for at least one patient [15].

Furthermore, participants claimed that they chose scientific peer-reviewed journals, and clinical guidelines from official bodies, conferences, and congresses as main sources of information, while it is worth noting that communication channels and business-to-customer marketing approaches are not well-developed in the field. These findings imply that physicians wish for more clinical evidence to prove PGx’s effectiveness along with more comprehensive clinical guidelines that would facilitate to implement PGx testing in clinical practice. Jia and coworkers (2022) concluded with similar results. In their study, respondents were informed about PGx testing via clinical guidelines and academic conferences [15]. In Deininger and coworkers, 2020, physicians reported to mainly getting informed by scientific literature (61.6%) following by discussions with peers. Less than 10% of participants declared to have used clinical guidelines as PGx resource [11]. Although, the existing literature focusing on the PGx source of information is limited, it is worth mentioning that searching on scientific literature is the main and most reliable source of information across the different countries while clinical guidelines are not so popular.

Respondents claimed that there are many stakeholders involved in the PGx testing supply chain including patients, physicians, PGx laboratories, pharmaceutical companies, and regulatory authorities. All these stakeholders interact with each other, but physicians are the interface of all. This observation agrees with Carroll and coworkers, 2016 study which highlighted that physicians had a central role, but they needed support from geneticists and genetic counselors, a comment that was made by our participants to [14]. In their study, Haga and coworkers (2012) claimed that physicians had the most responsibilities for the PGx process, in line with our results [12]. Deininger and coworkers (2019) shed light on another perspective about stakeholders by showing the need for interaction among all stakeholders [10]. Stakeholders’ relationships were in the scope of this study and agreed with the findings of other publications [27]. In general, all stakeholders mentioned by the interviewees are related to the field in Greece, a fact that is also supported by relevant and more specific research on the topic by Mitropoulou and coworkers (2014) [26].

PGx technology is an important innovation that can change the way patients’ therapeutic scheme is developed. According to this study’s findings, there are a few things that can play a significant role and are considered as change agents. Physicians are change agents because they directly interact with patients along with the rest key stakeholders. Laboratories, public authorities, health insurance (public & private) companies, and hospital managers are also motivated to be change factors since they are reducing spending and improving clinical outcomes. Finally, pharmaceutical companies are involved since their goal is to increase revenues and market shares. Therefore, any initiative to boost PGx adoption in clinical practice should consider the whole set of PGx change agents, and especially any potential deviations in their strategic goals. In many other studies, researchers illustrated that such resorts may lead to PGx advancement along with the reduction of PGx cost [28, 29].

All physicians were aware of the benefits of PGx testing in drug and disease management with a focus on its contribution to low risk of ADRs, better patient adherence to treatment, better clinical effectiveness, quality of life improvement, and reduction of healthcare expenditures. Our observations are congruent with the literature. Primary care mental health providers when interviewed by Vest and coworkers, 2020, stated the relative advantage of PGx testing and they were aware of all its clinical benefits [30]. Brazilian psychiatrists also noted that PGx can reduce the side effects of medications and help physicians choose the right medication for their patients in recent qualitative research [31]. In addition, based on Deininger and coworkers, 2020 survey, almost 60% of physicians agreed that PGx can lead to better clinical outcomes, while Lemke and coworkers, 2017, highlighted PGx benefits for patient experience in terms of their psychology, the reduction of time needed and the avoidance of medication titration that is associated with decreased healthcare expenses [11, 13].

Furthermore, lack of reimbursement, high PGx testing cost, lack of specialized personnel, and low level of physicians’ knowledge along the lengthy procedure of PGx order were the most important barriers found. Based on the study by Lau-Min and coworkers, 2022, oncologists pinpointed that the whole PGx service workflow was taking time and could be troublesome, while, Jia and coworkers, 2022, also pinpointed the scarcity of human resources, cost of PGx testing, and the limited physicians’ knowledge on the topic [15, 32]. According to Lemke and coworkers, 2017, physicians believe that high cost is the main burden and are worried that patients wouldn’t cover such costs unless it is important for their health [13].

In our study, specialists highlighted the cost as a challenging factor but were more optimistic that patients would pay for it. In Greece, the average cost of PGx testing is 150 euros and it is considered rather affordable, while in other countries might be higher [33]. For example, physicians in the United States underlined that most third-party vendors don’t reimburse such testing and the cost is relatively high for patients [7, 29]. Consequently, the pace of PGx incorporation into clinics can be slowed and varies among countries.

Participants were optimistic about the future of PGx technology in Greece. Most of them supported that PGx clinical applications will receive better adoption in the next years by the scientific community, especially physicians. Nonetheless, they clarified that pivotal interventions should be made in different aspects to allow the introduction of this innovation in clinical practice. Enhancing clinical research on the topic will allow professionals to gain insight into PGx effectiveness and it will provide valuable evidence to boost technology’s reimbursement by public bodies and subsequently PGx adoption. This fact is following the literature. Unertl and coworkers, 2015, stated that even if existing challenges and barriers are overcome shortly, more clinical evidence should be presented to support the need for PGx testing in patient management [34].

Moreover, we found that better physician and patient training will also be a decisive initiative along with an upgrade in the legal framework. Improving physicians’ education and knowledge in the field is also highlighted in the literature. Specifically, Koufaki and coworkers, 2023, have emphasized the positive impact of proper PGx training on future healthcare professionals and concluded that it can change the current status [35]. Schwartz and Issa, 2017, have clearly demonstrated the urgent need for a change in the legal and governmental framework of US towards PGx clinical applications [36]. Despite the fact that, in that study, researchers supported the role of pharmacists in PGx application, they have raised awareness about the launch of educational programs on the field to have well-trained healthcare professionals. In general, our study’s interviewees showed an interest in the PGx application and provided valuable feedback. They enlightened us about what society needs and what should be the next target in getting PGx testing reimbursed and widely adopted. As healthcare professionals and patients’ advocates, they shared insightful perceptions of the future of PGx.

### Limitations

This study has a few limitations. The study’s sample was limited in terms of number of respondents. However, the number of interviews in our research is quite similar to other relevant qualitative research designs and the key criterion employed to decide whether sufficient interviews have been undertaken was data saturation, namely when it was realized that the last few interviews provided no new themes and rather similar explanations. Additionally, semi-structured interviews were conducted in a cohort consisting of specialists with different levels of education, age, specialty, and years of experience. Moreover, no transcript application was used because of the discrepancies noticed in the translation.

## Conclusions

Overall, our findings bring out prominent aspects of the PGx testing scenario in Greece and ways to improve the future implementation of PGx. Physicians’ narratives provide valuable feedback that sheds light on the current situation in the country and give suggestions for the future. Besides the lack of experience with the use of PGx testing, physicians were aware of PGx clinical applications and advantages while they mentioned the most important barriers from their daily routine. In the future, PGx adoption will be wider in clinical practice thanks to different reasons. However, it was suggested that physicians improve their level of knowledge and expertise by pursuing PGx training. All specialists involved in the PGx process are vital to be ready to deal with PGx services and properly inform and motivate patients to conduct relevant testing.

### Electronic supplementary material

Below is the link to the electronic supplementary material.

Supplementary Material 1

## Data Availability

Data is provided within the manuscript or supplementary information files.

## Abbreviations

PGx: Pharmacogenomics
FDA: Food & Drug Associations
EMA: European Medication Association
CFIR: Consolidated Framework for Implementation Research
ADR: Adverse Drug Reactions
CPIC: Clinical Pharmacogenetics Implementation Consortium
DPWG: Dutch Pharmacogenetics Working Group
