# Supplementary material for: A qualitative approach to assess the opinion of physicians about the challenges and prospects of pharmacogenomic testing implementation in clinical practice in Greece
Source: Hum Genomics. 2024 Jul 19;18:82. doi: 10.1186/s40246-024-00648-y (PMC11264745; doi:10.1186/s40246-024-00648-y)
Supplement: Supplementary file 1 — Supplementary Material 1 [file 40246_2024_648_MOESM1_ESM.docx]

**A qualitative approach to assess the opinion of physicians about the challenges and future prospects of pharmacogenomic testing implementation in clinical practice**

**Semi-structured interview guide**

1. **Awareness & use of PGx testing**
   1. Have you attended any courses or lectures about PGx during your studies (undergraduate or postgraduate)?
   2. Have you ever recommended any PGx testing to a patient so far?
   3. Do you think that there are PGx applications available in your specialty?
   4. Do you know any colleague who has recommended any PGx testing so far?
   5. Are there any clinical guidelines for PGx implementation in everyday practice in your specialty?
2. **Sources of information** **about PGx**
   1. According to your opinion, what are the main (most valuable) sources/entities of information about PGx?
   2. How important do you think the above sources/entities are for informing/updating the various PGx stakeholders?
   3. Which types/channels of communication do these sources/entities use to contact (inform/update) their target stakeholders?
3. **Key stakeholders of the PGx supply chain and their interactions**
   1. Who are the main stakeholders in the PGx supply chain?
   2. What are the main characteristics (contribution) of each stakeholder in PGx testing implementation/adoption in clinical practice?
   3. How do stakeholders interact (cooperate) with each other to provide PGx testing to patients?
4. **Change agents** **in the PGx supply chain, i.e., those with a strong incentive (e.g., physicians, industries) to boost PGx adoption in daily clinical practice**
   1. What are the main change agents in the PGx supply chain?
   2. What are the change agents’ motives (main benefits) to boost PGx adoption in clinical practice?
   3. What is their power/strength/influence (e.g., bargaining, persuasion?) on other stakeholders to achieve the widespread PGx adoption in clinical practice?
5. **Benefits &** **usefulness of PGx testing**
   1. Which do you think that are the main benefits (motives) from the implementation of PGx testing ?
6. **Barriers of PGx implementation and Lack of reimbursement of PGx testing**
   1. Which are the main barriers that prevent physicians recommending PGx testing to their patients?
   2. PGx testing isn’t reimbursed in Greece by health insurance.
      1. To what extent and why (e.g., inability of the patient to pay, evidence that public authorities do not acknowledge PGx benefits as important? etc.) do you think that the lack of reimbursement may affect the widespread PGx adoption in everyday clinical practice?
      2. Which do you think are the main reasons for the lack of reimbursement of PGx tests in our country?
7. **Future prospects & Recommendations to boost the PGx adoption rate.**
   1. What do you think will be the most important advances/developments in PGx testing adoption in clinical practice in the next 10 years in Greece?
   2. How could the PGx adoption rate be ameliorated in Greece?
